# Tropical Diarrhœa

**Published:** 1890-09-13

**Authors:** 


### TROPICAL DIARRHŒA.

In the *Lancet* recently there was an article on the dietetic treatment of tropical diarrhœa by Dr. Neve, surgeon to the Mission Hospital, Kahsmir. Dr. Neve remarks, that he has recently seen many statements by eminent authorities, that milk and milk only must be given in tropical diarrhœa. The writer mentions a case of tropical diarrhœa in which the milk treatment had been pursued for more than a month the patient being in no way benefited. After coming under Dr. Neve's care, the same treatment was continued for three weeks longer without any advantage. Dr. Neve began therefore to think of sending the patient to a different climate, but before recommending this, resolved to try a change of diet, not to allow a diet of milk, but simple meat diet with toast and weak tea. Immediately this system was adopted the diarrhœa stopped. Dr. Neve says the change was suggested to him by the following sentence in Dr. Lauder Brunton's Croonian lecture of 1889: "Another mode of destroying the activity of bacteria is to starve them out by substituting a new diet for that to which they are accustomed." For this reason Vaughan also recommended that in infantile diarrhœa, beef-tea, rice-water, or pure water, should be given instead of milk for some days, until the bacteria may have died out. Now there is something attractive in the theory of starving out microbes. But the question may be fairly asked—are microbes the cause of tropical diarrhœa? This malady has been regarded as the consequence of change of climate from the lowlands to the elevated regions; or into the colder atmosphere of Europe from the tropics; or even from the lowering of temperature which takes place in the tropics on the advent of the cold season. This so called hill diarrhœa often commences in a few hours after a person has ascended from the Indian plains to the Indian hills, and it can scarcely be supposed that microbes so suddenly attack, especially in the purer air of the Indian mountains. Then again, if the malady persists certain changes of structure take place. The intestinal coats become atrophied and lardaceous and fatty deposits are found. All this is sufficient to cause diarrhœa without microbes.

Now as regards the milk treatment, Sir Joseph Fayrer must be regarded as the exponent, *vide* his article in the *Lancet* of last year. Sir Joseph Fayrer insists on the necessity of a milk diet, and there is no doubt that in many cases it is very beneficial. But the most recent author on Indian diseases, Sir William Moore, says an exclusively animal diet has been recommended but he prefers that patients should have what they have been accustomed to, which is usually a mixed diet; of course prohibiting irritating indigestible food. With regard to milk this writer reminds us that milk becomes a solid in the stomach, that it is not always well assimulated by diseased persons, and that some people cannot take it. If, however, certain precautions are attended to a milk diet often proves very beneficial, but it is not an universal panacea for chronic diarrhœa.

